# *N*′-(4-Chloro­benzyl­idene)-2-[4-(methyl­sulfan­yl)phen­yl]acetohydrazide

**DOI:** 10.1107/S1600536811039857

**Published:** 2011-10-05

**Authors:** Hoong-Kun Fun, Madhukar Hemamalini, V. Sumangala, D. Jagadeesh Prasad, Boja Poojary

**Affiliations:** a X-ray Crystallography Unit, School of Physics, Universiti Sains Malaysia, 11800 USM, Penang, Malaysia; b Department of Chemistry, Mangalore University, Mangalagangothri 574 199, Mangalore, Karnataka, India

## Abstract

In the title compound, C_16_H_15_ClN_2_OS, the hydrazine group is twisted slightly: the C—N—N—C torsion angle is 175.46 (13)°. The dihedral angle between the two terminal aromatic rings is 87.01 (8)°. In the crystal, inversion dimers linked by pairs of N—H⋯O hydrogen bonds generate *R*_2_^2^(8) loops. The dimers are further linked by weak C—H⋯π inter­actions.

## Related literature

For further details of aroyl­hydro­zones, see: Li & Qu (2011 ▶); Zhang (2011 ▶); Fan *et al.* (2010 ▶). Ajani *et al.* (2010 ▶); Avaji *et al.* (2009 ▶); Rasras *et al.* (2010 ▶). For graph-set notation, see: Bernstein *et al.* (1995 ▶).
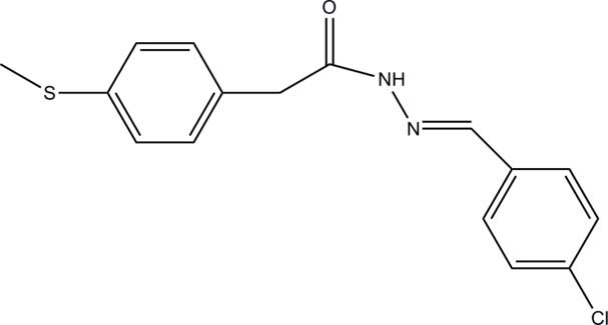


## Experimental

### 

#### Crystal data

C_16_H_15_ClN_2_OS
*M* *_r_* = 318.81
Monoclinic, 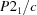

*a* = 17.0923 (13) Å
*b* = 9.6719 (7) Å
*c* = 9.5592 (7) Å
β = 92.399 (1)°
*V* = 1578.9 (2) Å^3^
*Z* = 4
Mo *K*α radiation
μ = 0.37 mm^−1^
*T* = 296 K
0.91 × 0.49 × 0.09 mm

#### Data collection

Bruker APEXII DUO CCD diffractometer
Absorption correction: multi-scan (*SADABS*; Bruker, 2009 ▶) *T*_min_ = 0.728, *T*_max_ = 0.967
17083 measured reflections
4748 independent reflections
3306 reflections with *I* > 2σ(*I*)
*R*_int_ = 0.025

#### Refinement

*R*[*F* ^2^ > 2σ(*F* ^2^)] = 0.041
*wR*(*F* ^2^) = 0.122
*S* = 1.04
4748 reflections
191 parameters
H-atom parameters constrained
Δρ_max_ = 0.41 e Å^−3^
Δρ_min_ = −0.41 e Å^−3^

### 

Data collection: *APEX2* (Bruker, 2009 ▶); cell refinement: *SAINT* (Bruker, 2009 ▶); data reduction: *SAINT*; program(s) used to solve structure: *SHELXTL* (Sheldrick, 2008 ▶); program(s) used to refine structure: *SHELXTL*; molecular graphics: *SHELXTL*; software used to prepare material for publication: *SHELXTL* and *PLATON* (Spek, 2009 ▶).

## Supplementary Material

Crystal structure: contains datablock(s) global, I. DOI: 10.1107/S1600536811039857/hb6422sup1.cif

Structure factors: contains datablock(s) I. DOI: 10.1107/S1600536811039857/hb6422Isup2.hkl

Supplementary material file. DOI: 10.1107/S1600536811039857/hb6422Isup3.cml

Additional supplementary materials:  crystallographic information; 3D view; checkCIF report

## Figures and Tables

**Table 1:** Hydrogen-bond geometry (Å, °) *Cg*1 and *Cg*2 are the centroids of the C1–C6 and C10–C15 rings, respectively.

*D*—H⋯*A*	*D*—H	H⋯*A*	*D*⋯*A*	*D*—H⋯*A*
N2—H1*N*2⋯O1^i^	0.95	2.03	2.9784 (17)	176
C14—H14*A*⋯*Cg*1^ii^	0.93	2.89	3.7627 (17)	156
C5—H5*A*⋯*Cg*2^iii^	0.93	2.98	3.4638 (17)	114

Symmetry codes: (i) 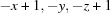; (ii) 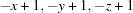; (iii) 
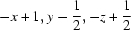
.

**Fig. 1.**
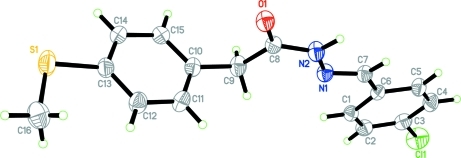
The asymmetric unit of the title compound, showing 50% probability displacement ellipsoids.

**Fig. 2.**
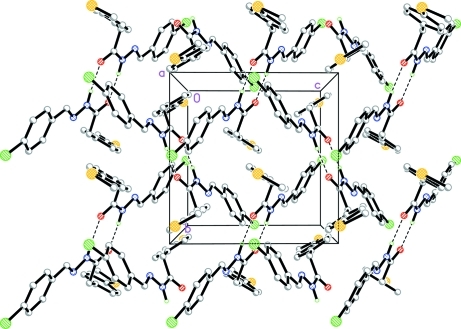
The crystal packing of the title compound (I). H atoms not involved in hydrogen bonding are omitted.

## supplementary crystallographic information

### Comment

Large number of aroylhydrozones have been synthesized in the recent years (Li & Qu, 2011; Zhang, 2011; Fan *et al.*, 2010) which can serve as intermediates in synthesizing biologically active compounds (Ajani *et al.*, 2010; Avaji *et al.*, 2009; Rasras *et al.*,  2010).

The asymmetric unit of the title compound is shown in Fig. 1. The hydrazine group is twisted slightly, with C7-N1-N2-C8, N1-N2-C8-C9 and N2-N1-C7-C6  torsion angles of 175.46 (13)°, 5.6 (2)° and -177.96 (12)°, respectively.  The dihedral angle between the two terminal (C1–C6/C10–C15) phenyl rings is 87.01 (8)°.

In the crystal structure, (Fig. 2),  centrosymmetrically related molecules are linked into dimers *via* pairs of intermolecular N2—H1N2···O1 (Table 1) hydrogen bonds, generating *R*_2_^2^(8) ring motifs (Bernstein *et al.*, 1995). The crystal structure is further stabilized by C—H···π interactions involving the centroids of the C1–C6 (Cg1) and C10–C15 (Cg2) rings.

### Experimental

An equimolar mixture of 2-(4-methylsulfanylphenyl)acetohydrazide and 4-chlorobenzaldehyde was refluxed for four hours in the presence of few drops of acid catalyst and ethanol as solvent.The compound obtained was filtered, washed, dried and recrystalised from ethanol to yield colourless plates.

### Refinement

All hydrogen atoms were positioned geometrically [N–H = 0.9458 Å and C–H = 0.93–0.97 Å] and were refined using a riding model, with *U*_iso_(H) = 1.2 or 1.5 *U*_eq_(C).  A rotating group model was applied to the methyl groups.

### Crystal data

**Table:**

C_16_H_15_ClN_2_OS	*F*(000) = 664
*M**_r_* = 318.81	*D*_x_ = 1.341 Mg m^−^^3^
Monoclinic, *P*2_1_/*c*	Mo *K*α radiation, λ = 0.71073 Å
Hall symbol: -P 2ybc	Cell parameters from 4828 reflections
*a* = 17.0923 (13) Å	θ = 3.0–29.5°
*b* = 9.6719 (7) Å	µ = 0.37 mm^−^^1^
*c* = 9.5592 (7) Å	*T* = 296 K
β = 92.399 (1)°	Plate, colourless
*V* = 1578.9 (2) Å^3^	0.91 × 0.49 × 0.09 mm
*Z* = 4	

### Data collection

**Table:**

Bruker APEXII DUO CCD diffractometer	4748 independent reflections
Radiation source: fine-focus sealed tube	3306 reflections with *I* > 2σ(*I*)
graphite	*R*_int_ = 0.025
φ and ω scans	θ_max_ = 30.3°, θ_min_ = 2.4°
Absorption correction: multi-scan (*SADABS*; Bruker, 2009)	*h* = −24→24
*T*_min_ = 0.728, *T*_max_ = 0.967	*k* = −13→13
17083 measured reflections	*l* = −9→13

### Refinement

**Table:**

Refinement on *F*^2^	Primary atom site location: structure-invariant direct methods
Least-squares matrix: full	Secondary atom site location: difference Fourier map
*R*[*F*^2^ > 2σ(*F*^2^)] = 0.041	Hydrogen site location: inferred from neighbouring sites
*wR*(*F*^2^) = 0.122	H-atom parameters constrained
*S* = 1.04	*w* = 1/[σ^2^(*F*_o_^2^) + (0.0504*P*)^2^ + 0.3224*P*] where *P* = (*F*_o_^2^ + 2*F*_c_^2^)/3
4748 reflections	(Δ/σ)_max_ < 0.001
191 parameters	Δρ_max_ = 0.41 e Å^−^^3^
0 restraints	Δρ_min_ = −0.41 e Å^−^^3^

### Special details

**Table:**

Geometry. All s.u.'s (except the s.u. in the dihedral angle between two l.s. planes) are estimated using the full covariance matrix. The cell s.u.'s are taken into account individually in the estimation of s.u.'s in distances, angles and torsion angles; correlations between s.u.'s in cell parameters are only used when they are defined by crystal symmetry. An approximate (isotropic) treatment of cell s.u.'s is used for estimating s.u.'s involving l.s. planes.
Refinement. Refinement of F^2^ against ALL reflections. The weighted R-factor wR and goodness of fit S are based on F^2^, conventional R-factors R are based on F, with F set to zero for negative F^2^. The threshold expression of F^2^ > 2σ(F^2^) is used only for calculating R-factors(gt) etc. and is not relevant to the choice of reflections for refinement. R-factors based on F^2^ are statistically about twice as large as those based on F, and R- factors based on ALL data will be even larger.

### Fractional atomic coordinates and isotropic or equivalent isotropic displacement parameters (Å^2^)

**Table:**

	*x*	*y*	*z*	*U*_iso_*/*U*_eq_	
Cl1	0.89531 (3)	0.48335 (6)	0.00009 (6)	0.0873 (2)	
S1	0.08573 (3)	0.40633 (6)	0.47773 (6)	0.07612 (17)	
O1	0.42041 (6)	0.12422 (11)	0.50622 (11)	0.0523 (3)	
N1	0.57757 (7)	0.23664 (12)	0.31358 (13)	0.0471 (3)	
N2	0.52912 (7)	0.15328 (13)	0.38944 (14)	0.0509 (3)	
H1N2	0.5458	0.0642	0.4186	0.061*	
C1	0.68893 (8)	0.38659 (15)	0.15181 (15)	0.0457 (3)	
H1A	0.6393	0.4255	0.1543	0.055*	
C2	0.74751 (9)	0.45572 (16)	0.08466 (16)	0.0525 (3)	
H2A	0.7375	0.5404	0.0414	0.063*	
C3	0.82114 (8)	0.39671 (17)	0.08305 (17)	0.0534 (4)	
C4	0.83721 (9)	0.27056 (18)	0.14429 (18)	0.0574 (4)	
H4A	0.8869	0.2318	0.1409	0.069*	
C5	0.77820 (8)	0.20250 (16)	0.21090 (17)	0.0509 (3)	
H5A	0.7885	0.1174	0.2531	0.061*	
C6	0.70357 (7)	0.25944 (14)	0.21570 (14)	0.0409 (3)	
C7	0.64405 (8)	0.18409 (14)	0.29029 (15)	0.0448 (3)	
H7A	0.6551	0.0949	0.3217	0.054*	
C8	0.45912 (7)	0.19818 (14)	0.43038 (15)	0.0429 (3)	
C9	0.43186 (8)	0.33897 (16)	0.37856 (19)	0.0542 (4)	
H9A	0.4615	0.4101	0.4289	0.065*	
H9B	0.4423	0.3476	0.2800	0.065*	
C10	0.34576 (8)	0.36138 (14)	0.39812 (16)	0.0468 (3)	
C11	0.29124 (9)	0.30706 (16)	0.30280 (18)	0.0566 (4)	
H11A	0.3085	0.2621	0.2238	0.068*	
C12	0.21138 (9)	0.31748 (17)	0.32120 (19)	0.0570 (4)	
H12A	0.1759	0.2789	0.2559	0.068*	
C13	0.18467 (8)	0.38616 (16)	0.43821 (17)	0.0508 (3)	
C14	0.23916 (9)	0.44558 (18)	0.53125 (16)	0.0557 (4)	
H14A	0.2221	0.4946	0.6079	0.067*	
C15	0.31841 (9)	0.43303 (17)	0.51179 (16)	0.0527 (3)	
H15A	0.3540	0.4732	0.5759	0.063*	
C16	0.03229 (11)	0.3267 (3)	0.3351 (3)	0.0869 (6)	
H16A	−0.0227	0.3311	0.3511	0.130*	
H16B	0.0480	0.2318	0.3277	0.130*	
H16C	0.0428	0.3744	0.2499	0.130*	

### Atomic displacement parameters (Å^2^)

**Table:**

	*U*^11^	*U*^22^	*U*^33^	*U*^12^	*U*^13^	*U*^23^
Cl1	0.0641 (3)	0.1044 (4)	0.0953 (4)	−0.0326 (3)	0.0244 (3)	0.0087 (3)
S1	0.0464 (2)	0.0932 (4)	0.0895 (4)	0.0137 (2)	0.0113 (2)	−0.0114 (3)
O1	0.0428 (5)	0.0508 (6)	0.0643 (7)	−0.0037 (4)	0.0130 (5)	0.0114 (5)
N1	0.0419 (6)	0.0468 (6)	0.0534 (7)	−0.0020 (5)	0.0129 (5)	0.0063 (5)
N2	0.0442 (6)	0.0456 (6)	0.0642 (8)	0.0015 (5)	0.0160 (5)	0.0132 (6)
C1	0.0417 (6)	0.0492 (8)	0.0466 (7)	0.0028 (5)	0.0053 (5)	0.0008 (6)
C2	0.0561 (8)	0.0507 (8)	0.0511 (8)	−0.0055 (7)	0.0068 (6)	0.0041 (6)
C3	0.0450 (7)	0.0645 (9)	0.0513 (8)	−0.0145 (7)	0.0091 (6)	−0.0050 (7)
C4	0.0384 (7)	0.0679 (10)	0.0665 (10)	0.0024 (7)	0.0094 (6)	−0.0055 (8)
C5	0.0453 (7)	0.0493 (8)	0.0587 (9)	0.0054 (6)	0.0099 (6)	0.0006 (7)
C6	0.0396 (6)	0.0431 (7)	0.0402 (7)	−0.0001 (5)	0.0061 (5)	−0.0034 (5)
C7	0.0445 (7)	0.0419 (7)	0.0486 (8)	0.0006 (5)	0.0076 (6)	0.0021 (6)
C8	0.0384 (6)	0.0435 (7)	0.0472 (7)	−0.0051 (5)	0.0064 (5)	0.0006 (6)
C9	0.0457 (7)	0.0458 (8)	0.0722 (10)	−0.0010 (6)	0.0166 (7)	0.0080 (7)
C10	0.0454 (7)	0.0394 (7)	0.0563 (8)	0.0023 (5)	0.0108 (6)	0.0070 (6)
C11	0.0557 (8)	0.0532 (8)	0.0619 (10)	0.0023 (7)	0.0143 (7)	−0.0126 (7)
C12	0.0505 (8)	0.0545 (9)	0.0659 (10)	0.0017 (7)	0.0031 (7)	−0.0119 (7)
C13	0.0457 (7)	0.0502 (8)	0.0569 (9)	0.0093 (6)	0.0067 (6)	0.0044 (7)
C14	0.0541 (8)	0.0666 (10)	0.0468 (8)	0.0132 (7)	0.0065 (6)	−0.0047 (7)
C15	0.0504 (8)	0.0575 (9)	0.0501 (8)	0.0046 (6)	0.0007 (6)	−0.0003 (7)
C16	0.0516 (10)	0.1089 (18)	0.0998 (16)	−0.0040 (10)	−0.0003 (10)	−0.0019 (13)

### Geometric parameters (Å, °)

**Table:**

Cl1—C3	1.7386 (15)	C7—H7A	0.9300
S1—C13	1.7590 (15)	C8—C9	1.516 (2)
S1—C16	1.783 (2)	C9—C10	1.5069 (19)
O1—C8	1.2310 (16)	C9—H9A	0.9700
N1—C7	1.2728 (17)	C9—H9B	0.9700
N1—N2	1.3828 (15)	C10—C11	1.380 (2)
N2—C8	1.3462 (17)	C10—C15	1.386 (2)
N2—H1N2	0.9458	C11—C12	1.387 (2)
C1—C2	1.3840 (19)	C11—H11A	0.9300
C1—C6	1.391 (2)	C12—C13	1.394 (2)
C1—H1A	0.9300	C12—H12A	0.9300
C2—C3	1.383 (2)	C13—C14	1.385 (2)
C2—H2A	0.9300	C14—C15	1.380 (2)
C3—C4	1.376 (2)	C14—H14A	0.9300
C4—C5	1.382 (2)	C15—H15A	0.9300
C4—H4A	0.9300	C16—H16A	0.9600
C5—C6	1.3920 (18)	C16—H16B	0.9600
C5—H5A	0.9300	C16—H16C	0.9600
C6—C7	1.4612 (18)		
			
C13—S1—C16	104.73 (9)	C10—C9—H9A	109.2
C7—N1—N2	114.68 (12)	C8—C9—H9A	109.2
C8—N2—N1	121.57 (12)	C10—C9—H9B	109.2
C8—N2—H1N2	117.9	C8—C9—H9B	109.2
N1—N2—H1N2	120.4	H9A—C9—H9B	107.9
C2—C1—C6	120.69 (13)	C11—C10—C15	117.86 (13)
C2—C1—H1A	119.7	C11—C10—C9	119.97 (14)
C6—C1—H1A	119.7	C15—C10—C9	122.16 (14)
C3—C2—C1	118.83 (14)	C10—C11—C12	121.98 (14)
C3—C2—H2A	120.6	C10—C11—H11A	119.0
C1—C2—H2A	120.6	C12—C11—H11A	119.0
C4—C3—C2	121.80 (14)	C11—C12—C13	119.56 (15)
C4—C3—Cl1	119.02 (12)	C11—C12—H12A	120.2
C2—C3—Cl1	119.18 (13)	C13—C12—H12A	120.2
C3—C4—C5	118.81 (14)	C14—C13—C12	118.61 (14)
C3—C4—H4A	120.6	C14—C13—S1	116.28 (12)
C5—C4—H4A	120.6	C12—C13—S1	125.11 (13)
C4—C5—C6	120.94 (14)	C15—C14—C13	120.94 (14)
C4—C5—H5A	119.5	C15—C14—H14A	119.5
C6—C5—H5A	119.5	C13—C14—H14A	119.5
C1—C6—C5	118.92 (13)	C14—C15—C10	120.97 (15)
C1—C6—C7	122.62 (12)	C14—C15—H15A	119.5
C5—C6—C7	118.45 (13)	C10—C15—H15A	119.5
N1—C7—C6	122.07 (13)	S1—C16—H16A	109.5
N1—C7—H7A	119.0	S1—C16—H16B	109.5
C6—C7—H7A	119.0	H16A—C16—H16B	109.5
O1—C8—N2	119.34 (13)	S1—C16—H16C	109.5
O1—C8—C9	123.30 (12)	H16A—C16—H16C	109.5
N2—C8—C9	117.36 (12)	H16B—C16—H16C	109.5
C10—C9—C8	112.16 (11)		
			
C7—N1—N2—C8	175.46 (13)	O1—C8—C9—C10	−14.8 (2)
C6—C1—C2—C3	−0.5 (2)	N2—C8—C9—C10	164.74 (14)
C1—C2—C3—C4	1.1 (2)	C8—C9—C10—C11	−81.06 (19)
C1—C2—C3—Cl1	−179.53 (11)	C8—C9—C10—C15	97.43 (17)
C2—C3—C4—C5	−1.0 (2)	C15—C10—C11—C12	−2.7 (2)
Cl1—C3—C4—C5	179.58 (12)	C9—C10—C11—C12	175.83 (15)
C3—C4—C5—C6	0.4 (2)	C10—C11—C12—C13	0.8 (3)
C2—C1—C6—C5	−0.1 (2)	C11—C12—C13—C14	1.7 (2)
C2—C1—C6—C7	178.68 (14)	C11—C12—C13—S1	−179.14 (13)
C4—C5—C6—C1	0.1 (2)	C16—S1—C13—C14	177.21 (14)
C4—C5—C6—C7	−178.68 (14)	C16—S1—C13—C12	−1.93 (18)
N2—N1—C7—C6	−177.96 (12)	C12—C13—C14—C15	−2.3 (2)
C1—C6—C7—N1	−7.3 (2)	S1—C13—C14—C15	178.46 (13)
C5—C6—C7—N1	171.47 (14)	C13—C14—C15—C10	0.4 (2)
N1—N2—C8—O1	−174.81 (13)	C11—C10—C15—C14	2.1 (2)
N1—N2—C8—C9	5.6 (2)	C9—C10—C15—C14	−176.42 (14)

### Hydrogen-bond geometry (Å, °)

**Table:**

Cg1 and Cg2 are the centroids of the C1–C6 and C10–C15 rings, respectively.

**Table:**

*D*—H···*A*	*D*—H	H···*A*	*D*···*A*	*D*—H···*A*
N2—H1N2···O1^i^	0.95	2.03	2.9784 (17)	176
C14—H14A···Cg1^ii^	0.93	2.89	3.7627 (17)	156
C5—H5A···Cg2^iii^	0.93	2.98	3.4638 (17)	114

Symmetry codes: (i) −*x*+1, −*y*, −*z*+1; (ii) −*x*+1, −*y*+1, −*z*+1; (iii) −*x*+1, *y*−1/2, −*z*+1/2.
